# The Role of Sex Hormone-Binding Globulin (SHBG) as a Marker of Metabolic Dysfunction-Associated Steatotic Liver Disease, with an Extended Analysis in Both Men and Women

**DOI:** 10.3390/jcm15031301

**Published:** 2026-02-06

**Authors:** Ljiljana Fodor Duric, Zrinka Čolak Romić, Dino Pavičić, Josip Čurić, Velimir Belčić, Ivija Rajković, Irijana Rajković, Jelena Muslim, Nikolina Basic Jukic, Bozidar Vujicic, Tonko Gulin, Matko Gulin, Mladen Grgurević, Anja Oberiter Korbar

**Affiliations:** 1 School of Medicine, University of Catholica Croatica, 10000 Zagreb, Croatia; 2 Department of Neurology, University Hospital Dubrava, 10000 Zagreb, Croatia; 3 Medikol Polyclinic, 10000 Zagreb, Croatiajosipcuric875@gmail.com (J.Č.); vebelcic@gmail.com (V.B.); jelena.muslim@medikol.hr (J.M.); 4 Department of Nephrology, Arterial Hypertension, Dialysis and Transplantation, University Hospital Centre, 10000 Zagreb, Croatia; 5 School of Medicine, University of Zagreb, 10000 Zagreb, Croatia; 6 Department of Nephrology, Dialysis and Kidney Transplantation, University Hospital Centre Rijeka, 51000 Rijeka, Croatia; 7 School of Medicine, University of Rijeka, 51000 Rijeka, Croatia; 8 Department of Nephrology, Arterial Hypertension and Dialysis, University Hospital Centre “Sestre Milosrdnice”, 10000 Zagreb, Croatia; 9 Department of Radiology, University Hospital Centre “Sestre Milosrdnice”, 10000 Zagreb, Croatia; 10 Department of Diabetes, Endocrinology and Metabolic Diseases, Vuk Vrhovac, Merkur University Hospital, 10000 Zagreb, Croatia; 11 CAIR-Centar, The House of Statistics, 10000 Zagreb, Croatia; anja.oberiter-korbar@cair-center.hr

**Keywords:** MASLD, sex hormone-binding globulin (SHBG), insulin resistance, metabolic disturbances, sex differences

## Abstract

**Introduction**: Metabolic dysfunction-associated steatotic liver disease (MASLD) is associated with insulin resistance and metabolic disturbances. Sex hormone-binding globulin (SHBG) is closely linked to metabolic regulation and has been shown to differ between individuals with and without MASLD. **Objective**: This study aimed to investigate the associations between SHBG and MASLD and their relationships with insulin resistance, body mass index (BMI), age, and sex in a combined male–female cohort. **Patients and Methods**: We studied 98 men and 54 women with MASLD and 74 men and 55 women without MASLD (aged 25–64 years). Participants underwent abdominal ultrasonography and fasting blood sampling, including measurements of glucose, liver enzymes, lipids, insulin, SHBG, estradiol, and testosterone. **Results**: SHBG levels were lower in individuals with MASLD than in controls, with a more pronounced reduction in women. MASLD status was associated with an approximately 10 nmol/L lower SHBG concentration (*p* < 0.0001; gender × MASLD interaction *p* = 0.0462). Higher estradiol levels were associated with higher SHBG concentrations (*p* = 0.0009), although this association differed by sex (gender × log-estradiol interaction *p* = 0.0147). Older age and higher total cholesterol levels were associated with higher SHBG levels, whereas higher triglyceride levels were associated with lower SHBG levels. **Conclusions**: SHBG showed significant associations with MASLD and with key metabolic and hormonal factors, including BMI, age, and sex. Inclusion of both men and women extends prior male-only research and provides a broader characterisation of sex-specific associations in MASLD.

## 1. Introduction

Fatty liver, or metabolic dysfunction-associated steatotic liver disease (MASLD), is currently one of the most common chronic liver diseases in the general population [1,2]. Its prevalence continues to rise, particularly in the context of the global epidemic of obesity, insulin resistance, and metabolic syndrome. Although MASLD has long been considered a metabolically driven liver disease, accumulating evidence suggests that hormonal factors are associated with its presence and metabolic profile [2,3,4].

Sex hormone-binding globulin (SHBG) is a glycoprotein that binds sex hormones, regulates their bioavailability, and has been widely studied as a marker of metabolic health [5,6]. Lower SHBG concentrations have consistently been linked to insulin resistance, type 2 diabetes, and cardiovascular diseases. Several observational studies have also reported associations between lower SHBG levels and the presence of fatty liver detected by imaging methods [7,8,9,10,11,12].

In our previous study, conducted exclusively in a male population, we demonstrated a significant association between lower SHBG concentrations and MASLD, independent of several metabolic parameters [1]. However, the absence of female participants limited the generalizability of those findings. Given the well-documented sex differences in SHBG concentrations, sex hormone levels, and metabolic characteristics, inclusion of women is necessary for a more comprehensive assessment of the relationship between SHBG and MASLD.

Therefore, the present study aimed to extend our previous research to a combined male–female cohort undergoing routine health examinations, with fatty liver identified by ultrasonography. We examined associations among SHBG, metabolic variables, and sex hormones in relation to MASLD, with particular attention to sex-specific patterns and interactions among hormonal and metabolic parameters.

## 2. Materials and Methods

This study included 281 participants. In the MASLD group, 98 men and 54 women were diagnosed with metabolic dysfunction-associated steatotic liver disease based on qualitative abdominal ultrasonography, with ages ranging from 29 to 73 years. The control group consisted of 74 men and 55 women without fatty liver on ultrasonography, aged 25 to 64 years.

Inclusion criteria for the MASLD group were age 29–73 years, elevated body mass index (BMI), and ultrasound-confirmed fatty liver. Inclusion criteria for the control group were age 25–64 years and the absence of fatty liver on ultrasound.

Exclusion criteria included ongoing treatment for malignant diseases and a diagnosis of type 2 diabetes. Alcohol consumption and physical activity were assessed anamnestically; participants reported moderate alcohol intake and low levels of physical activity. As these characteristics did not differ substantially between groups, they were not presented in tabular form. All participants had normal fasting glucose values, and individuals with diabetes were excluded to minimise potential metabolic confounding.

Ultrasound examinations were performed using a Siemens Acuson Sequoia ultrasound system. MASLD was diagnosed qualitatively based on increased liver echogenicity, without specific grading of steatosis severity, as standardised ultrasound criteria for precise classification are not established. Patients with MASLD underwent abdominal ultrasound with an extended protocol due to increased waist circumference, while healthy controls underwent standard abdominal ultrasound. For a more detailed assessment of fatty liver, future studies could incorporate elastography or FibroScan.

Blood samples were collected to measure biochemical, hormonal, and metabolic parameters, including estradiol, testosterone, and sex hormone-binding globulin (SHBG). Estradiol and testosterone were assessed at a single time point, and their temporal variability was not addressed in this study.

Enrollment for the study occurred between January 2024 and August 2025. The study was conducted in accordance with the Declaration of Helsinki and approved by the Institutional Ethical Board of Medikol Polyclinic on 28 October 2025 (approval number 01.01.024 26/22-13). Informed consent was obtained from all participants before their inclusion in the study.

## 3. Results

### 3.1. Statistical Analysis

To investigate the role of SHBG (sex hormone-binding globulin) in MASLD and to identify determinants of MASLD (sex hormone-binding globulin), a series of complementary statistical approaches was applied. First, descriptive statistics were used to summarise the distribution of clinical, biochemical, and hormonal variables in the study population, providing an overview of central tendencies and variability and establishing baseline characteristics of participants with and without fatty liver. A *t*-test was then performed among women to evaluate whether hormonal or metabolic parameters differed significantly between affected and unaffected individuals, and to allow comparison with results from the corresponding analysis previously conducted in men.

Subsequently, two general linear models (GLMs), a complete and a stepwise model, were applied to assess associations between continuous variables and SHBG as the outcome measure. These models allowed adjustment for potential confounding factors and enabled the formal testing of interaction effects, which were further illustrated using interaction plots [13,14]. Logistic regression analyses were then performed to model the probability of fatty liver as a binary outcome. Univariate and bivariate models were used to evaluate the independent effects of individual factors and to explore specific interactions. Finally, a multivariate logistic regression model was constructed to jointly examine metabolic and hormonal factors in relation to MASLD, allowing assessment of their combined associations with the presence of fatty liver [15,16]. This multivariate modelling strategy was undertaken primarily for exploratory and explanatory purposes, to characterise patterns of association, rather than to develop a predictive model intended for clinical application.

Statistical analyses were performed using SAS software, version 9.4 (SAS Institute Inc., Cary, NC, USA).

#### 3.1.1. Descriptive Characteristics of the Study Population

Univariate summaries of numeric variables, stratified by gender (men/women) and MASLD status (healthy vs. fatty liver), are presented in Table 1. Descriptive statistics summarise central tendency and variability and inform subsequent modelling; no inferential comparisons are included at this stage. For each stratum, Table 1 shows the number of observations (N), the mean (SD), the median, and the range (minimum and maximum values). Overall, participants with fatty liver exhibited higher central values of liver enzymes (ALT, AST, GGT), adiposity markers (BMI, waist circumference, weight), fasting insulin and HOMA2, triglycerides, and CRP, along with lower HDL, in both men and women. Total cholesterol and LDL levels showed less consistent differences across groups.

Fasting glucose medians were generally similar between groups, although upper ranges were wider among participants with fatty liver. SHBG levels were lower in the fatty liver groups for both men and women. In men, total testosterone was lower among those with fatty liver, while in women, free testosterone tended to be higher in the fatty liver group. Estradiol distributions were similar between groups in men, whereas in women, estradiol was markedly lower in the fatty liver group and showed a right-skewed distribution, particularly among healthy women. Age differed between the female groups (mean 44.0 vs. 53.3 years for healthy and fatty liver groups, respectively), which may partially explain the observed estradiol differences.

However, data on menopausal status and hormone therapy were not available, which should be considered when interpreting these descriptive summaries. Distributions of estradiol (pmol/L), insulin, triglycerides, CRP, and GGT were right-skewed, as indicated by larger SDs, differences between means and medians, and coefficients of skewness. Creatinine values were within expected gender-specific ranges.

#### 3.1.2. Comparison of Individuals Diagnosed with Fatty Liver to Healthy Participants About SHBG, Separately by Gender

In our earlier article [1], *t*-test results indicated a statistically significant difference in SHBG levels between men with fatty liver and healthy participants. We now apply the same *t*-test framework to the female cohort to evaluate whether analogous differences are present in women.

The table below (Table 2) displays the mean SHBG difference (along with the respective 95% Confidence Interval (CI) limits) between MASLD groups for women (−22.75, 95% CI −15.21, −30.29) and compares it to the previously reported estimates (−9.51, 95% CI −5.23, −13.79) for male participants (Table 3). Group differences were tested using the Welch–Satterthwaite *t*-test (Table 4 and Table 5 for women and men, respectively).

Among women, mean SHBG concentrations were markedly lower in participants with fatty liver than in the healthy ones (44.01, 95% CI 38.59, 49.43 vs. 66.76, 95% CI 61.40, 72.13), yielding a mean difference of −22.75 (with 95% CI limits of −15.21 to −30.29) and a *p*-value of less than 0.0001. In men, the same pattern was observed but with smaller absolute values: fatty liver 29.14 (95% CI 26.88, 31.41) vs. healthy 38.65 (95% CI 34.99, 42.32), for a mean difference of −9.51 (95% CI −5.23, −13.79), also statistically significant. Comparing genders, women have higher SHBG than men within each of the MASLD strata, and the difference in SHBG between MASLD groups in women (−22.75) is roughly 2.4-fold to that in men (−9.51); notably, the 95% confidence intervals for these gender-specific differences do not overlap (women −15.21, −30.29; men −5.23, −13.79), indicating a clearly stronger contrast in women.

**Figure 1:**
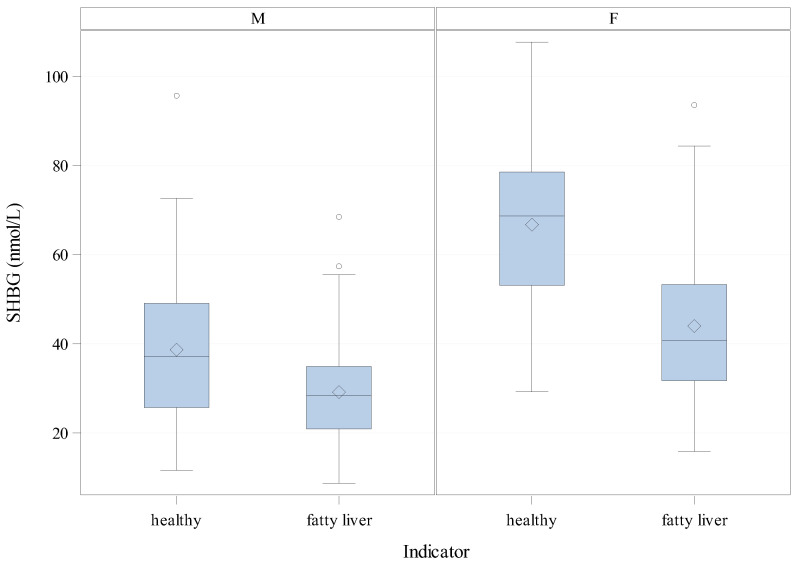
Boxplots visualise the distributions and between-group contrasts.

The distributions and between-group contrasts are visualised in the boxplots below (Figure 1), which highlight medians, interquartile ranges, and potential outliers, allowing a side-by-side appraisal of SHBG patterns for women (F) versus men (M).

#### 3.1.3. General Linear Model Analysis for SHBG

SHBG was analysed as the outcome in a combined male–female cohort using a general linear model (GLM). Two strategies were applied: (i) a complete model including all candidate variables without automated selection, and (ii) a stepwise selection approach (using the SAS GLMSELECT procedure with the Schwarz Bayesian Information Criterion (SBC) as the stopping criterion). To better meet model assumptions, variables with right-skewed distributions (AST, CRP, GGT, glucose, triglycerides, HOMA2, and estradiol) were log-transformed before the analysis [17].

Results for the full model are displayed in Table 6.

As shown in Table 6, the MASLD Indicator (fatty liver) was associated with lower SHBG concentrations (*p* = 0.0024), indicating that individuals with fatty liver had lower SHBG than healthy controls, after adjustment for all other variables in the model. Gender was a strong determinant, with women exhibiting markedly higher levels than men (*p* = 0.0003). Among metabolic parameters, total cholesterol was positively associated with SHBG (*p* = 0.0025), while log-transformed triglycerides showed a negative relationship (*p* = 0.0202), highlighting opposing lipid effects.

Hormonal variables were also relevant. Log-transformed estradiol was positively associated with SHBG (*p* = 0.0076), and a significant log (Estradiol) × gender interaction (*p* = 0.0311) indicated that the strength of this association differed by gender. Specifically, increases in estradiol were associated with greater increases in SHBG in men than in women, suggesting gender-specific slopes.

Other variables, including BMI, age, BMI × gender, BMI × age, log (HOMA2), HDL cholesterol, creatinine, log (AST), log (CRP), log (GGT), log (Glucose), and free testosterone, were not significantly associated with SHBG after mutual adjustment (all *p* > 0.05). Although age was included as a covariate in general linear models and thus adjusted for, this adjustment does not fully account for unmeasured reproductive or hormonal factors influencing estradiol levels, leaving the possibility of residual confounding.

These findings indicate that fatty liver, gender, cholesterol, triglycerides, and estradiol (with a gender interaction) are the principal independent determinants of SHBG levels. In contrast, other metabolic and biochemical variables do not contribute significantly to the whole model. Because tests of individual covariates are conditional on the remaining terms in the model, their statistical significance in the complete model may be affected by substantial correlations among the included measures.

Therefore, stepwise selection of model covariates was performed using the Schwarz Bayesian Information Criterion (SBC) as the stopping criterion.

The chosen model results are presented in Table 7, below (General Linear Model—Parameter Estimates for the Chosen Model). After adjustment for all covariates, SHBG levels differed by gender (*p* = 0.0018), with higher levels in women than men (by 80 nmol/L on average, regardless of the fatty liver status). MASLD Indicator (fatty liver) was associated with lower SHBG (by 10 nmol/L) compared with healthy status (*p* < 0.0001), and this reduction was more pronounced in women (gender × MASLD Indicator *p* = 0.0462). (On average, this reduction in SHBG was by 10 nmol/L higher in women than in men.) The association between estradiol (log-transformed) and SHBG also varied by gender, being weaker among women than men (gender × log (Estradiol) *p* = 0.0147). Furthermore, higher estradiol (log-transformed) was associated with higher SHBG (*p* = 0.0009). Overall, older age and higher total cholesterol were associated with higher SHBG (*p* = 0.0058 and *p* = 0.0003, respectively), whereas higher triglycerides (log-transformed) were associated with lower SHBG (*p* = 0.0012).

#### 3.1.4. Association of Metabolic and Hormonal Factors with the MASLD Status Using Logistic Regression

To investigate the metabolic and hormonal determinants of MASLD status, logistic regression models of increasing complexity were applied: from univariate to bivariate models incorporating key interactions, and culminating in a multivariate model. This sequential approach allowed us first to assess the independent associations of individual measures with the MASLD indicator, then examine potential gender-specific hormonal effects, and finally jointly evaluate metabolic and endocrine factors within a comprehensive modelling framework. Further details regarding variable selection approaches are provided in the Supplementary Materials.

In the univariate analyses (Table 8 (1. Univariate) and Figure S1), body mass index (BMI), cholesterol, and SHBG each showed significant associations with the MASLD status. Specifically, every two-unit increase in BMI was associated with an approximate fourfold increase in the odds of fatty liver (OR = 4.070, 95% CI: 3.021–5.804). These results should be interpreted as statistical associations rather than causal effects, consistent with the study’s cross-sectional design.

**Figure 2:**
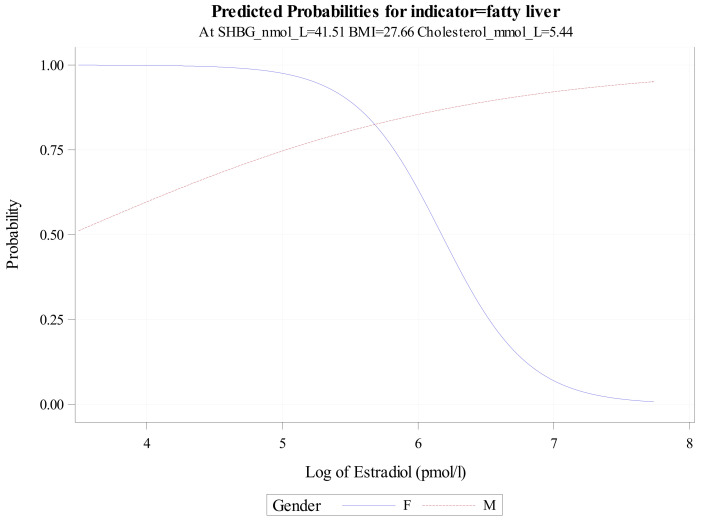
Fitted associations between log-transformed estradiol and MASLD from the bivariate logistic regression model, stratified by gender.

The bivariate logistic regression model, including an interaction between gender and log-transformed estradiol (Table 8 (2. Bivariate); Figure 2), demonstrated a sex-specific association pattern. Among women, a 0.693-unit increase in log-transformed estradiol concentration (corresponding to a doubling of estradiol levels) was associated with lower odds of the outcome (OR = 0.152, 95% CI: 0.070–0.273). In men, estradiol concentration was not significantly associated with the outcome, with point estimates in the opposite direction (OR = 1.513, 95% CI: 0.697–3.340). These estimates reflect cross-sectional associations and should not be interpreted as evidence of causality. Information on menopausal status, hormone replacement therapy, and oral contraceptive use was not available.

Figure 2 presents the fitted associations between log-transformed estradiol and the outcome, stratified by gender. The crossing of the curves reflects the inclusion of a gender-by-estradiol interaction term. It is shown to visualise the modelled associations rather than to imply a biologically meaningful threshold, directional effect, or individual-level risk.

**Figure 3:**
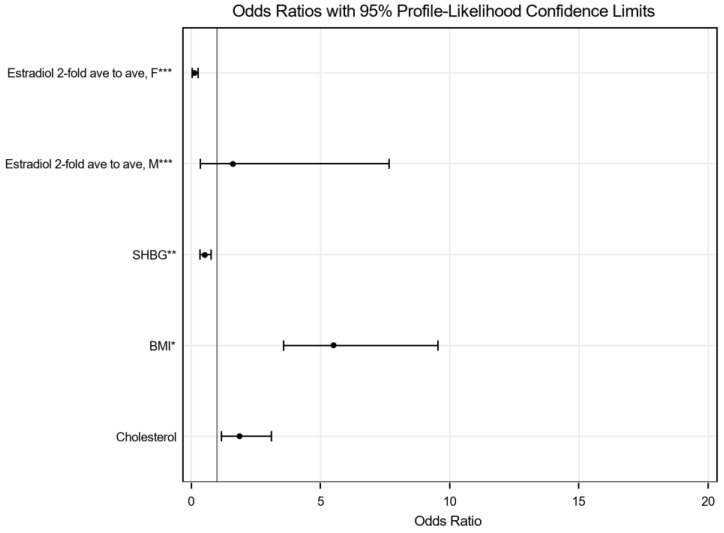
Forest Plot (odds ratios with 95% profile likelihood confidence intervals). * BMI odds ratio corresponds to a 2-point increase in BMI. ** SHBG odds ratio corresponds to a 10 nmol/L increase in SHBG. *** Estradiol odds ratio corresponds to a 2-fold increase in estradiol.

The final multivariable logistic regression model results are shown in Table 8 (3. Multivariate) and Figure 3. Body mass index (BMI; per 2-unit increase) showed the strongest association with MASLD (OR = 5.508, 95% CI: 3.574–9.545), followed by total cholesterol (OR = 1.864, 95% CI: 1.174–3.104). Sex hormone-binding globulin (SHBG; per 10 nmol/L increase) was associated with lower odds of MASLD (OR = 0.521, 95% CI: 0.331–0.764).

Estradiol showed a sex-specific association pattern. In women, higher estradiol concentrations were associated with lower odds of MASLD (OR = 0.113, 95% CI: 0.031–0.265 for a two-fold increase), whereas no statistically significant association was observed in men (OR = 1.612, 95% CI: 0.354–7.656). These estimates represent cross-sectional associations and should not be interpreted as evidence of causality.

The multivariable model was developed for exploratory, hypothesis-generating purposes rather than for clinical prediction. Accordingly, it should not be interpreted as a clinically applicable predictive model. The use of multivariable analysis reflects the joint evaluation of correlated metabolic and hormonal factors and does not imply definitive predictive accuracy.

#### 3.1.5. Association of BMI, Blood Pressure, and Gender with MASLD/Fatty Liver Indicator

In this expanded cohort, including both men and women, the larger sample size provided greater statistical power to detect associations between blood pressure and MASLD Indicator (Table 9). In the univariate analyses, BMI, diastolic blood pressure, and systolic blood pressure were all strongly associated with MASLD Indicator. BMI showed the most robust effect (Wald χ^2^ = 71.7986, *p* < 0.0001), followed by systolic blood pressure (Wald χ^2^ = 31.8567, *p* < 0.0001) and diastolic blood pressure (Wald χ^2^ = 23.2789, *p* < 0.0001). Gender was not significantly associated with MASLD Indicator when examined univariately (Wald χ^2^ = 1.4822, *p* = 0.2234).

The bivariate models further clarified these associations. When BMI and diastolic blood pressure were entered together, BMI remained highly significant (Wald χ^2^ = 67.9669, *p* < 0.0001). In contrast, diastolic blood pressure lost significance (Wald χ^2^ = 3.5003, *p* = 0.0614), due to a strong correlation among BMI, diastolic and systolic blood pressure. In the model including BMI and systolic blood pressure, BMI retained a strong association (Wald χ^2^ = 65.4434, *p* < 0.0001), while systolic blood pressure showed a modest but significant effect (Wald χ^2^ = 5.4772, *p* = 0.0193).

In the final multivariate model including BMI, gender, and diastolic and systolic blood pressure, BMI remained the variable most strongly associated with fatty liver (Wald χ^2^ = 64.4213, *p* < 0.0001). Neither diastolic blood pressure (Wald χ^2^ = 0.9434, *p* = 0.3314) nor systolic blood pressure (Wald χ^2^ = 2.8172, *p* = 0.0933) remained significant after mutual adjustment, and gender was not associated with MASLD Indicator (Wald χ^2^ = 0.0066, *p* = 0.9351).

These findings demonstrate that, in the larger mixed-gender cohort, BMI showed the strongest and most consistent association with fatty liver. In contrast, diastolic and systolic blood pressure and gender were not significantly associated after mutual adjustment.

## 4. Discussion

In this cross-sectional study, we investigated the associations of SHBG, estradiol, and metabolic factors with MASLD in a combined male and female cohort.

Consistent with previous findings in men, SHBG levels were significantly lower in individuals with fatty liver. Extending the analysis to women revealed even stronger contrasts, with women showing a roughly 2.4-fold greater difference in SHBG levels between MASLD patients and healthy participants than men. These findings highlight the importance of considering sex-specific hormonal and metabolic influences in MASLD.

Estradiol demonstrated a potentially protective association in women, whereas no significant relationship was observed in men. This supports the concept that sex hormones may modulate MASLD pathophysiology in a sex-specific manner, particularly in women. Our results align with previous studies reporting associations between lower SHBG concentrations, altered sex hormone profiles, metabolic dysregulation, and hepatic steatosis.

A notable limitation of this study is the significant age difference observed between female MASLD patients and female controls. The MASLD group was, on average, nearly a decade older, suggesting a higher proportion of postmenopausal women. The menopausal transition is associated with substantial changes in oestrogen levels, SHBG concentrations, and metabolic risk profiles, all of which may influence SHBG levels and MASLD development. As direct data on menopausal status were unavailable, this potential confounding factor could not be fully adjusted for, and should be carefully considered when interpreting sex-specific associations in our results.

Given the cross-sectional design, no causal or mechanistic conclusions can be drawn, and the observed associations do not allow inference regarding directionality. In particular, alterations in hepatic metabolic function associated with MASLD may influence SHBG synthesis, rather than SHBG changes contributing causally to disease development.

BMI emerged as the most robust predictor of MASLD, independent of sex and blood pressure. While systolic and diastolic blood pressure were initially associated with MASLD, these associations were attenuated after adjustment for BMI, suggesting that adiposity mediates much of the cardiovascular contribution to fatty liver risk. Lipid parameters, including cholesterol and triglycerides, also influenced SHBG levels and MASLD risk, underscoring a complex interplay between lipid metabolism, hormonal status, and hepatic fat accumulation.

The strong predictive performance of the multivariate stepwise logistic regression model, incorporating metabolic and hormonal variables, demonstrates the potential value of integrative approaches for MASLD risk assessment. However, the absence of an independent validation cohort warrants caution, and external validation in separate populations is needed to confirm generalizability.

These findings have several potential clinical implications. SHBG and estradiol may serve as adjunct biomarkers for identifying individuals at higher risk of MASLD, particularly women, whereas BMI remains the primary modifiable risk factor. Interventions targeting weight management and metabolic health are likely to represent the most effective strategies for MASLD prevention.

Future research should aim to validate these associations in larger, multi-ethnic cohorts, assess longitudinal predictive value, and further explore mechanistic pathways linking sex hormones, SHBG, and hepatic lipid accumulation. Such studies could contribute to individualised risk stratification and inform targeted therapeutic approaches.

## 5. Conclusions

Our results confirm that lower SHBG levels are associated with MASLD in both men and women, with more potent effects in women. Estradiol shows a sex-specific association in women, whereas BMI remains the dominant metabolic determinant of fatty liver. Integrating metabolic and hormonal biomarkers may help in MASLD risk assessment and inform individualised prevention strategies. Future studies should focus on validation, mechanistic insights, and the development of gender-specific interventions to reduce the risk of fatty liver.

## Figures and Tables

**Table 1:** Descriptive statistics for men and women.

Gender	MASLD Indicator	N Obs	Variable	Mean	Std Dev	Median	Minimum	Maximum
M	healthy	74.00	ALT (U/L)	28.01	11.26	25.00	12.00	56.00
			AST (U/L)	26.41	9.76	24.00	16.00	73.00
			GGT (U/L)	22.41	16.98	17.50	6.00	115.00
			Body Mass Index (kg/m^2^)	23.53	1.36	23.65	18.90	26.90
			Waist Circumference (cm)	78.80	8.50	79.00	58.00	94.00
			Weight (kg)	77.82	7.74	78.00	60.00	102.00
			Height (cm)	181.69	6.86	182.00	168.00	205.00
			Glucose (mmol/L)	5.53	0.65	5.50	4.50	9.70
			Insulin (pmol/L)	62.01	30.63	52.80	14.10	167.00
			Triglycerides (mmol/L)	1.34	0.76	1.15	0.40	5.20
			Cholesterol (mmol/L)	5.25	1.05	5.05	3.00	8.90
			HDL (mmol/L)	1.36	0.34	1.30	0.80	2.20
			LDL (mmol/L)	3.31	0.87	3.25	1.70	6.40
			Creatinine (µmol/L)	91.46	10.27	90.00	56.00	115.00
			HOMA2	1.18	0.57	1.03	0.38	3.14
			C-Reactive Protein (mg/L)	1.77	2.68	0.85	0.20	17.10
			SHBG (nmol/L)	38.65	15.80	37.10	11.60	95.70
			Testosterone (nmol/L)	18.96	6.42	18.31	7.48	38.55
			Free Testosterone (pmol/L)	368.03	101.17	344.55	166.70	610.70
			Estradiol (pmol/L)	120.74	30.90	116.00	62.00	193.00
			Age	42.53	7.12	42.50	30.00	57.00
	fatty liver	98.00	ALT (U/L)	42.28	28.10	37.00	13.00	210.00
			AST (U/L)	31.15	18.98	27.00	15.00	173.00
			GGT (U/L)	49.98	50.69	34.00	7.00	362.00
			Body Mass Index (kg/m^2^)	30.48	3.91	30.20	21.80	47.32
			Waist Circumference (cm)	104.96	11.17	104.00	85.00	160.00
			Weight (kg)	100.70	14.57	100.00	69.00	160.00
			Height (cm)	181.54	7.57	180.00	166.00	200.00
			Glucose (mmol/L)	5.77	0.83	5.65	4.60	11.40
			Insulin (pmol/L)	102.82	60.79	90.15	21.50	413.00
			Triglycerides (mmol/L)	2.12	1.51	1.70	0.40	9.60
			Cholesterol (mmol/L)	5.64	0.99	5.50	3.00	8.10
			HDL (mmol/L)	1.21	0.30	1.20	0.60	2.40
			LDL (mmol/L)	3.69	0.95	3.50	1.50	6.30
			Creatinine (µmol/L)	94.21	11.85	93.00	68.00	124.00
			HOMA2	1.95	1.09	1.75	0.40	7.09
			C-Reactive Protein (mg/L)	2.48	2.81	1.60	0.10	19.00
			SHBG (nmol/L)	29.14	11.30	28.40	8.70	68.50
			Testosterone (nmol/L)	15.13	5.22	14.42	5.25	30.93
			Free Testosterone (pmol/L)	333.87	97.61	322.85	117.20	729.50
			Estradiol (pmol/L)	126.79	35.40	121.50	52.00	226.00
			Age	45.87	6.81	46.00	30.00	62.00
F	Healthy	55.00	ALT (U/L)	20.95	11.54	18.00	9.00	70.00
			AST (U/L)	22.40	5.93	21.00	14.00	54.00
			GGT (U/L)	17.87	14.86	13.00	9.00	91.00
			Body Mass Index (kg/m^2^)	23.99	3.26	23.80	17.60	34.20
			Waist Circumference (cm)	83.39	6.57	82.00	71.00	100.00
			Weight (kg)	67.62	9.34	65.00	53.00	93.00
			Height (cm)	168.02	6.55	168.00	152.00	180.00
			Glucose (mmol/L)	5.31	0.61	5.20	4.60	8.20
			Insulin (pmol/L)	61.62	21.67	65.10	21.90	114.30
			Triglycerides (mmol/L)	1.07	0.56	0.90	0.50	3.20
			Cholesterol (mmol/L)	5.21	0.93	5.00	3.80	8.30
			HDL (mmol/L)	1.73	0.35	1.70	1.20	2.80
			LDL (mmol/L)	2.98	0.90	2.70	1.60	5.60
			Creatinine (µmol/L)	71.85	9.35	73.00	50.00	96.00
			HOMA2	1.17	0.41	1.20	0.40	2.10
			C-Reactive Protein (mg/L)	1.61	2.97	0.80	0.20	21.20
			SHBG (nmol/L)	66.76	19.08	68.70	29.20	107.70
			Testosterone (nmol/L)	1.22	0.59	1.36	0.12	2.21
			Free Testosterone (pmol/L)	12.34	8.07	9.70	0.70	40.40
			Estradiol (pmol/L)	643.45	354.34	587.00	56.00	1610.00
			Age	44.04	9.40	44.00	25.00	64.00
	fatty liver	54.00	ALT (U/L)	31.24	15.42	26.50	15.00	84.00
			AST (U/L)	27.26	9.02	24.50	17.00	64.00
			GGT (U/L)	27.00	25.61	18.00	9.00	137.00
			Body Mass Index (kg/m^2^)	31.49	6.17	30.75	23.10	50.30
			Waist Circumference (cm)	85.36	8.44	89.00	74.00	105.00
			Weight (kg)	88.76	19.33	86.00	62.00	137.00
			Height (cm)	167.65	5.94	168.00	156.00	180.00
			Glucose (mmol/L)	5.62	0.84	5.60	2.50	7.40
			Insulin (pmol/L)	123.41	69.24	118.65	1.40	394.20
			Triglycerides (mmol/L)	1.79	0.76	1.65	0.60	4.10
			Cholesterol (mmol/L)	5.60	0.95	5.70	3.40	8.20
			HDL (mmol/L)	1.41	0.36	1.40	0.90	2.30
			LDL (mmol/L)	3.52	0.93	3.60	1.60	6.30
			Creatinine (µmol/L)	71.56	9.37	71.50	51.00	92.00
			HOMA2	2.42	1.22	2.20	0.50	7.40
			C-Reactive Protein (mg/L)	4.45	5.01	2.70	0.80	30.00
			SHBG (nmol/L)	44.01	19.86	40.70	15.80	93.60
			Testosterone (nmol/L)	0.97	0.63	0.91	0.09	2.73
			Free Testosterone (pmol/L)	15.83	12.40	13.00	1.20	55.20
			Estradiol (pmol/L)	148.83	107.24	99.50	47.00	516.00
			Age	53.30	9.26	54.00	29.00	73.00

**Table 2:** *t*-test results—mean and 95% confidence interval estimates (women).

MASLD Indicator	N	Mean	95% CL Mean		Std Dev	Std Err
fatty liver	54	44.0143	38.5948	49.4337	19.8553	2.7020
healthy	51	66.7627	61.3955	72.1300	19.0834	2.6722
Diff (1–2)		−22.7485	−15.2117	−30.2853		3.8002

**Table 3:** *t*-test results—mean and 95% confidence interval estimates (men).

MASLD Indicator	N	Mean	95% CL Mean		Std Dev	Std Err
fatty liver	98	29.1418	26.8760	31.4077	11.3017	1.1416
healthy	74	38.6541	34.9924	42.3158	15.8049	1.8373
Diff (1–2)		−9.5122	−5.2316	−13.7929		2.1631

**Table 4:** *t*-test results—Welch–Satterthwaite *t*-test (women).

DF	t Value	Pr > |t|
102.97	−5.99	<0.0001

**Table 5:** *t*-test results—Welch–Satterthwaite *t*-test (men).

DF	t Value	Pr > |t|
126.11	−4.40	<0.0001

**Table 6:** General Linear Model—Parameter Estimates for the Full Model.

Parameter	Estimate	Standard Error	t Value	Pr > |t|
Intercept	−23.374	39.932	−0.59	0.5588
MASLD Indicator: fatty liver	−11.322	3.699	−3.06	0.0024
MASLD Indicator: healthy	0.000			
Gender F	99.867	27.293	3.66	0.0003
Gender M	0.000			
log (Estradiol)	12.816	4.766	2.69	0.0076
BMI	−1.020	0.997	−1.02	0.3068
Age	−0.506	0.563	−0.9	0.3696
Cholesterol (mmol/L)	3.348	1.096	3.05	0.0025
log (Homa2)	−1.561	2.111	−0.74	0.4603
HDL	−0.474	3.417	−0.14	0.8897
Creatinine	−0.040	0.089	−0.45	0.6523
log (AST)	−2.862	3.251	−0.88	0.3795
log (CRP)	−1.193	1.076	−1.11	0.2685
log (GGT)	1.717	1.829	0.94	0.3489
log (Glucose)	2.156	9.233	0.23	0.8155
log (Triglycerides)	−5.738	2.455	−2.34	0.0202
Free Testosterone	0.012	0.014	0.88	0.3773
Gender F * MASLD Indicator:fatty liver	−10.024	6.588	−1.52	0.1294
Gender F * MASLD Indicator:healthy	0.000			
Gender M * MASLD Indicator:fatty liver	0.000			
Gender M * MASLD Indicator: healthy	0.000			
log (Estradiol) * Gender F	−11.932	5.504	−2.17	0.0311
log (Estradiol) * Gender M	0.000			
BMI * Gender F	−0.595	0.513	−1.16	0.247
BMI * Gender M	0.000			
BMI * Age	0.030	0.020	1.5	0.1353

* Interaction between variables

**Table 7:** General Linear Model—Parameter Estimates for the Chosen Model.

Parameter	Estimate	Standard Error	t Value	Pr > |t|
Intercept	−62.603	21.46	−2.92	0.0038
MASLD Indicator: fatty liver	−10.039	2.463	−4.08	<0.0001
MASLD Indicator: healthy	0.000			
Gender F	79.594	25.255	3.15	0.0018
Gender M	0.000			
log (Estradiol)	14.399	4.273	3.37	0.0009
Age	0.336	0.121	2.78	0.0058
Cholesterol	3.721	1.01	3.68	0.0003
log (Triglycerides)	−6.552	1.997	−3.28	0.0012
Gender F * MASLD Indicator: fatty liver	−10.051	5.019	−2	0.0462
Gender F * MASLD Indicator: healthy	0.000			
Gender M * MASLD Indicator: fatty liver	0.000			
Gender M* MASLD Indicator: healthy	0.000			
Gender * log (Estradiol) F	−11.957	4.868	−2.46	0.0147
Gender * log (Estradiol) M	0.000			

* Interaction between variables

**Table 8:** Odds Ratio Estimates with Profile Likelihood Confidence Interval Limits for Univariate, Bivariate and Multivariate Models.

	Analysis								
	1. Univariate			2. Bivariate			3. Multivariate		
Effect	Odds Ratio Estimate	Profile Likelihood Lower Confidence Limit	Profile Likelihood Upper Confidence Limit	Odds Ratio Estimate	Profile Likelihood Lower Confidence Limit	Profile Likelihood Upper Confidence Limit	Odds Ratio Estimate	Profile Likelihood Lower Confidence Limit	Profile Likelihood Upper Confidence Limit
BMI *	4.070	3.021	5.804				5.508	3.574	9.545
Cholesterol	1.509	1.180	1.954				1.864	1.174	3.104
SHBG **	0.656	0.565	0.752				0.521	0.331	0.764
Estradiol 2-fold ave to ave, F ***				0.152	0.070	0.273	0.113	0.031	0.265
Estradiol 2-fold ave to ave, M ***				1.513	0.697	3.340	1.612	0.354	7.656

* BMI odds ratio corresponds to a 2-point increase in BMI. ** SHBG odds ratio corresponds to a 10 nmol/L increase in SHBG. *** Estradiol odds ratio corresponds to a 2-fold increase in estradiol.

**Table 9:** Wald Chi-Square statistics and *p*-values from univariate, bivariate, and multivariate logistic regression models for factors (BMI, diastolic, systolic and gender) of MASLD/fatty liver.

	Analysis							
	Univariate		Bivariate (Diastolic, Gender)		Bivariate (Systolic, Gender)		Multivariate	
	Wald χ^2^	Pr > χ^2^	Wald χ^2^	Pr > χ^2^	Wald χ^2^	Pr > χ^2^	Wald χ^2^	Pr > χ^2^
BMI	71.7986	<0.0001	67.9669	<0.0001	65.4434	<0.0001	64.4213	<0.0001
Diastolic	23.2789	<0.0001	3.5003	0.0614			0.9434	0.3314
Gender	1.4822	0.2234					0.0066	0.9351
Systolic	31.8567	<0.0001			5.4772	0.0193	2.8172	0.0933

## Data Availability

The original contributions presented in this study are included in the article. Further inquiries can be directed to the corresponding author.

## Supplementary Materials

The following supporting information can be downloaded at: https://www.mdpi.com/article/10.3390/jcm15031301/s1, Figure S1: Fitted associations between MASLD and (a) BMI, (b) SHBG, and (c) cholesterol. Figure S2: ROC curve for the chosen model. Table S1: Bootstrap validation.
