# Supplementary material for: The Role of Sex Hormone-Binding Globulin (SHBG) as a Marker of Metabolic Dysfunction-Associated Steatotic Liver Disease, with an Extended Analysis in Both Men and Women
Source: J Clin Med. 2026 Feb 6;15(3):1301. doi: 10.3390/jcm15031301 (PMC12897918; doi:10.3390/jcm15031301)
Supplement: Supplementary file 1 [file jcm-15-01301-s001.zip › jcm-4036470-supplementary.pdf]

Supplementary Materials

The Role of Sex Hormone-Binding Globulin (SHBG) as a Marker of Metabolic Dysfunction-Associated Steatotic Liver Disease, with an Extended Analysis in Both Men and Women

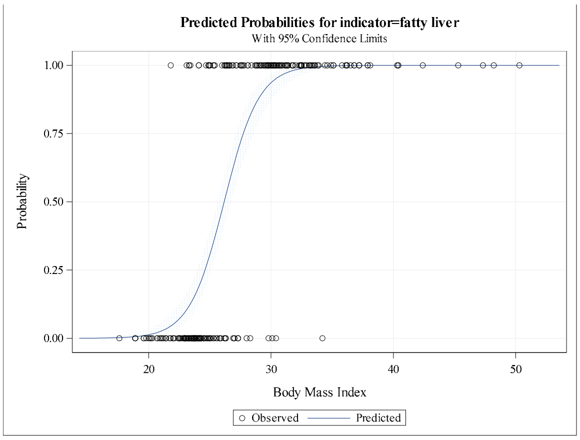

(**a**)

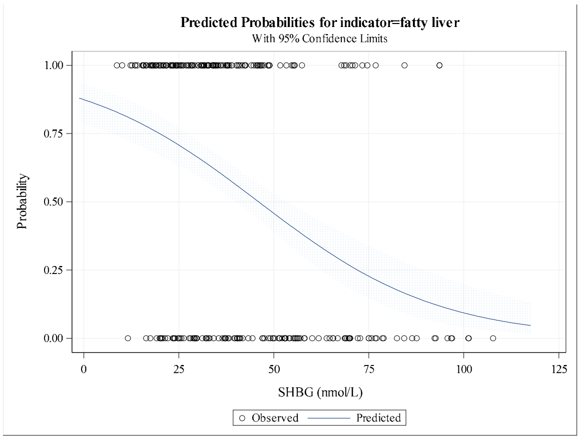

(**b**)

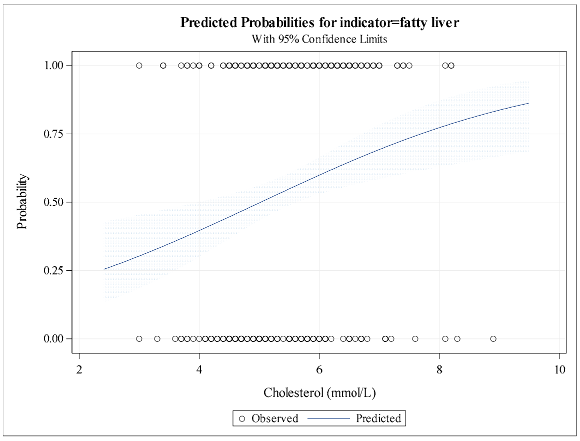

(**c**)

Figure S1. Fitted associations between MASLD and (a) BMI, (b) SHBG, and (c) cholesterol.

Figure S1 illustrates the associations between BMI, cholesterol, SHBG, and MASLD observed in the regression analyses. BMI showed a strong positive association with MASLD, with higher BMI values associated with higher odds of fatty liver. Total cholesterol was also

positively associated with MASLD (OR = 1.509, 95% CI: 1.180–1.954). In contrast, SHBG demonstrated an inverse association with MASLD, with higher SHBG levels associated with lower odds of fatty liver (OR = 0.656, 95% CI: 0.565–0.752 per 10 nmol/L increase).

The fitted curves shown in Figure S1 visualize these modeled associations across the observed ranges of the variables. All findings reflect cross-sectional statistical associations and should not be interpreted as causal effects.

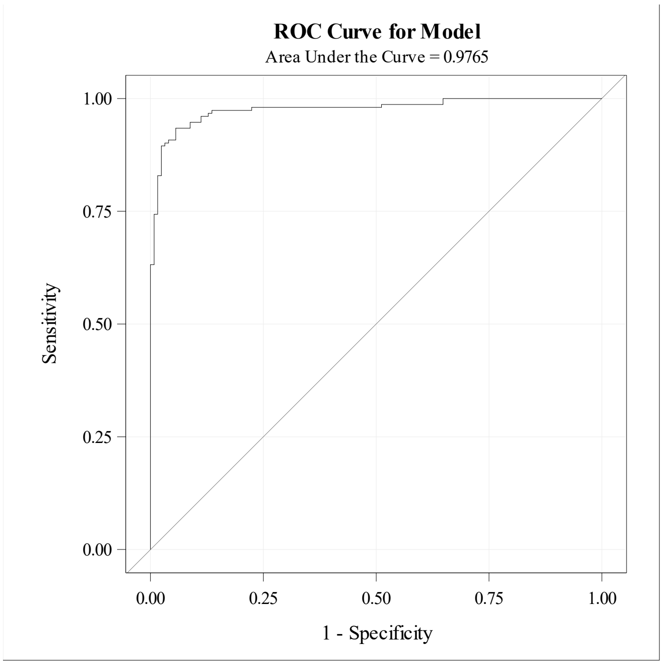

Figure S2. ROC curve for the final model.

Table S1. Bootstrap validation.

Naive AUC-Statistic	Optimism	Optimism-Corrected AUC-Statistic
0.976526	.006105685	0.97042

Multivariable Model Specification, Discrimination, and Internal Validation

Multivariable logistic regression models were constructed using a stepwise selection procedure, with model selection based on minimization of the Schwarz Bayesian Information Criterion (SBC). The SBC was used to balance model fit and complexity and to guide variable inclusion; discrimination metrics were not used to select or optimize the model.

Model discrimination was subsequently assessed descriptively using the area under the receiver operating characteristic curve (AUC). The AUC was reported to characterize within-sample model behavior and was not intended to establish predictive accuracy or clinical utility.

To assess potential overfitting, internal validation was performed using bootstrap resampling (1,000 iterations). The optimism-corrected AUC was compared with the apparent AUC to evaluate the stability of discrimination estimates within the available dataset. In addition, the dataset was randomly partitioned into training (60%) and validation (40%) subsets, and AUC values were calculated separately in each subset. These procedures were undertaken to explore model stability under resampling and data partitioning and do not constitute external validation.

Given the observational study design, modest sample size, and use of stepwise variable selection, all discrimination metrics should be interpreted with caution. In particular, stepwise selection may increase the risk of overfitting and model instability, potentially inflating apparent discrimination measures even when internal validation approaches are applied. Sensitivity analysis, using alternative variable selection approaches, was conducted.

The apparent AUC of the selected multivariable model was 0.976 (Figure S2). Following bootstrap-based internal validation, the optimism-corrected AUC was 0.970 (Table S1). In the random training–validation split, the AUC decreased from 0.982 in the training subset to 0.953 in the validation subset. These findings indicate relatively stable discrimination within the study sample but should not be interpreted as evidence of generalizable predictive performance. Importantly, discrimination metrics were evaluated after model selection and were not used to guide model building. We also emphasize that body mass index (BMI) alone demonstrated substantial discriminatory ability in this dataset (reaching an AUC value of 0.970).

Sensitivity Analyses Using Alternative Variable Selection Approaches

As a sensitivity analysis, additional multivariable logistic regression models were explored using alternative variable selection approaches, including forward selection, backward elimination, and score-based selection using the branch-and-bound algorithm of Furnival and Wilson

(1974). These analyses were conducted on the validation dataset to reduce the influence of variable selection and model fitting on the same data used to evaluate discrimination.

The purpose of these analyses was to assess whether the observed model discrimination was specific to the stepwise selection procedure or persisted across different selection strategies when evaluated in data not used for model construction. Across all approaches, discrimination estimates were similar, with AUC values consistently ranging between approximately 0.95 and 0.97. These findings indicate that the high discrimination observed is not unique to the stepwise approach but reflects separation between groups within the dataset.

Consistent with the exploratory nature of the study, these analyses were not intended to optimize model performance or to support predictive or clinical applicability. All discrimination metrics are reported for descriptive purposes only and should be interpreted with caution given the observational study design and sample size.
